# Responses of Broiler Chickens to Diets of Different Nutritional Planes Supplemented With or Without Organic Acids

**DOI:** 10.1002/vms3.70332

**Published:** 2025-04-07

**Authors:** Kolade M. Ogunola, A. V. Jegede, Adeboye O. Fafiolu, Oyegunle E. Oke

**Affiliations:** ^1^ Department of Animal Nutrition Federal University of Agriculture Abeokuta Abeokuta Nigeria; ^2^ Animal Physiology Department Federal University of Agriculture Abeokuta Abeokuta Nigeria; ^3^ Centre of Excellence in Avian Sciences University of Lome Lome Togo

**Keywords:** broiler, diets, environment, organic acid, performance

## Abstract

**Background:**

Scientific information on the diets of various nutritional planes supplemented with organic acids is scarce in tropical environments.

**Objectives:**

This study was conducted to evaluate the response of broilers to diets of different nutritional planes supplemented with or without organic acids. A total of 240 one‐day‐old unsexed broiler chicks of the Cobb 500 strain were randomly assigned to 6 treatments, each with 4 replicates having 10 chicks.

**Methods:**

The treatments were chickens fed an optimal diet (optimal energy and protein) (Diet 1), a medium diet (medium energy and protein) (Diet 2) and a low diet (low energy and protein) (Diet 3), while diets 4, 5 and 6 were diets 1, 2 and 3 supplemented with 4, 6 and 8 g/kg organic acid (*Fysal‐MP*), respectively. The study was laid out in a completely randomized design.

**Results:**

The birds fed diets with organic acid supplementation recorded similar (*p *> 0.05) final weights compared to those fed an optimal diet (T1); however, the weights were higher (*p <* 0.05) compared to the chickens on a low diet without organic acid (T3). The feed conversion ratio was better in birds fed diets containing organic acid, irrespective of the nutritional plane. Birds on medium diets with organic acids recorded a higher (*p <* 0.05) aspartate aminotransferase compared to those on low diets during the starter phase. Birds on a low diet without organic acid recorded a lower (*p <* 0.05) basal width compared to those on other diets. The microbial counts were reduced (*p <* 0.05) in broilers fed organic acids, with the lowest reduction in the group fed medium diets with 6 g/kg organic acids.

**Conclusions:**

It was concluded that organic acid supplementation enhanced blood parameters, carcass traits, growth performance and microbial counts in broiler chickens.

## Graphical abstract

The study evaluated the response of broilers to diets of different nutritional planes supplemented with or without organic acids.

Two hundred and forty broiler chicks were used.

Different diet types were supplemented with 4, 6 and 8 g/kg organic acid.

Organic acid supplementation had beneficial impacts on broilers.

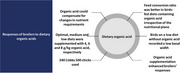

## 1 Introduction

Advancements in breeding, changes in climatic and other environmental conditions and daily increases in the price of feed ingredients necessitate a consistent evaluation of the nutrient requirements of chickens. The Intergovernmental Panel on Climate Change has reported that many nations are experiencing an increase in the frequency of climate‐related hazards like cyclones, floods, heat waves and droughts, with disproportionate effects on the poor and vulnerable populations in developing nations (IPCC 2014). Climate variability has significantly encumbered poultry production in the tropics (Onagbesan et al. 2023; Oke et al. 2024; Fushai et al. 2025).

The major needs of modern broiler industries are improved production and feed efficiency, which could be attained through specific feed additives (Oke et al. 2017; Oke 2018; Tokofai et al. 2020; Tokofai et al. 2021; Oke et al. 2021; Akosile, Sogunle, et al. 2023; Akosile, Majekodunmi et al. 2023; Kpomasse, Kouame, et al. 2023). In the commercial poultry industry, profit can be maximized when the birds have a healthy gastrointestinal tract, indicating that a healthy intestinal villus promotes nutrition absorption (Orbugh et al. 2024; Kpomasse, Oso et al. 2023; Oyelola et al. 2024). When high‐quality feed is provided and the intestinal villus is compromised by pathogenic bacteria toxins, the necessary feed efficiency cannot be reached, which results in a loss of profit (Zulfqarul et al. 2017). Rezaei et al. (2004) reported that feeding optimum nutrient levels reduced the feed cost and environmental pollution, improved the feed conversion ratio and growth rate and maximized the economic profit for broiler production.

Several feed additives like organic acids, prebiotics, symbiotics, probiotics and herbal growth promoters are known to have favourable effects in controlling pathogenic microorganisms and enhancing beneficial microorganism growth (Oke et al. 2024b). Dietary organic acids, among these additives, have drawn increased attention due to their antibacterial action and the ability to generate a reduced pH in the gut. This can enhance nutrient absorption in poultry diets (Partanen 2001; Kil et al. 2011; Cheng et al. 2014). Organic acid supplementation in feed enhances the growth performance of broiler chickens by improving nutrient absorption and decreasing the pathogenic bacteria population in the digestive tract (Adil et al. 2010).

Inorganic and organic acids are the two categories of dietary acids used in poultry. They are frequently included in poultry diets as a supplement. Carboxylic acids, including fatty acids, have the chemical structure of R‐COOH and are called organic acids. Because of their physiochemical characteristics, only short‐chain fatty acids like butyric acid (C4), propionic (C3), acetic (C2), formic (C1) and other carboxylic acids like citric acid, fumaric, tartaric, lactic and malic have been used most frequently in the poultry industry for various beneficial effects (Dibner and Winter 2002; Adil et al. 2010; Adhikari et al. 2020; Mustafa et al. 2021; Khan et al. 2022).

Like other agricultural activities, climate change and terror attacks negatively impact the supply of essential feed ingredients to poultry; hence, there is a need to focus on the effective utilization of the available feedstuff to reduce the cost of production. According to the report of Rezaei et al. (2004), feeding broiler birds with optimum nutrient levels minimized the cost of production and gave maximum growth performance with minimum cost. If feed efficiency can be improved, fewer nutrients will be required to attain the optimal nutrient requirement of chickens. There is, however, a dearth of information on the diets of different nutritional planes supplemented with organic acid. Therefore, the focus of this study was to slightly reduce the protein and energy requirement in the diets of broiler chickens and supplement such diets with organic acids; it is hypothesized that the improvement in feed utilization occasioned by organic acid supplementation could compensate for the slight reduction in protein and energy requirement, thus improving production at a reduced cost of feed. This study, therefore, evaluated the responses of broilers to diets of suboptimal nutritional planes supplemented with or without organic acids.

## 2 Materials and Methods

### 2.1 Experimental Materials

The additive used in this experiment was an organic acid called Fysal‐MP. The Fysal‐MP (organic acid) was manufactured by Selko International Netherlands, and it contained E280 propionic acid, E270 lactic acid, E260 acetic acid, E236 formic acid, E200 sorbic acid, E295 ammonium formate and E330 citric acid with a recommended dosage of 4 g/kg. The feedstuffs used were obtained from a commercial feed mill.

### 2.2 Experimental Birds, Management

This study involved the use of 240 one‐day‐old Cobb 500 strain broiler chicks assigned to 6 treatments of 40 birds each, having 4 replicates of 10 chickens per replicate. The birds were sourced from a reliable hatchery for the study. The building used for the study was an open‐sided poultry house, which was divided into 24 equal pens using wire mesh. The pen dimensions were (2 m long × 1 m wide). The brooding pen was cleaned and fumigated thoroughly 2 weeks prior to the commencement. The drinkers and feeders were also washed thoroughly and disinfected. On arrival, the birds were supplied with feed and water that contained vitamins as anti‐stress for 7 days. All other required routine management practices and medication schedules were performed throughout the 56 days of the study. The duration of the experiment was partitioned into two phases: the starter phase, lasting from 0 to 28 days of age, and 28 to 56 days of age as the finisher phase. Water and experimental feed were supplied without restriction.

### 2.3 Experimental Diets

Three diets (mash) were formulated such that Diet 1 was the control diet containing 23% CP (crude protein) and 2891 kcal/kg, Diets 2 and 3 contained suboptimal levels of 21% CP, 2759.90 kcal/kg ME (metabolizable energy) and 19% CP, 2600 kcal/kg ME, respectively, while Diets 4, 5 and 6 were Diets 1, 2 and 3 supplemented with 4, 6 and 8 g/kg organic acid, respectively (starter phase). The finisher phase diets were made so that Diet 1 had 21% CP and 3005.80 kcal/kg ME (control diet), Diets 2 and 3 contained suboptimal levels of 19% CP, 2871.20 kcal/kg ME and 17% CP, 2728.20 kcal/kg ME, respectively, while diets 4, 5 and 6 were diets 1, 2 and 3 supplemented with 4, 6 and 8 g/kg organic acid, respectively. Tables 1 and 2 show the experimental diets’ composition.

**TABLE 1:** Gross composition (%) of experimental diets (0–4 weeks).

	Diet					
Ingredient	1 Opt	2 Med	3 Low	4 +4 g/kg	5 +6 g/kg	6 +8 g/kg
Maize	52.00	46.00	40.00	52.00	46.00	40.00
Fishmeal	3.00	3.00	3.00	3.00	3.00	3.00
Soya meal	30.00	24.00	21.00	30.00	24.00	21.00
Groundnut cake	6.00	5.00	4.00	6.00	5.00	4.00
Wheat offal	3.00	16.00	26.00	3.00	16.00	26.00
Bone meal	3.00	3.00	3.00	3.00	3.00	3.00
Oyster shell	2.00	2.00	2.00	2.00	2.00	2.00
Lysine	0.25	0.25	0.25	0.25	0.25	0.25
Methionine	0.25	0.25	0.25	0.25	0.25	0.25
Premix^a^	0.25	0.25	0.25	0.25	0.25	0.25
Salt (NaCl)	0.25	0.25	0.25	0.25	0.25	0.25
Total	100.00	100.00	100.00	100.00	100.00	100.00
Calculated analysis						
M.E (kcal/kg)	2891.0	2759.90	2600.00	2891.00	2759.90	2600.00
CP (%)	23.10	21.29	19.38	23.10	21.29	19.38
Fat (%)	3.75	3.72	3.68	3.75	3.72	3.68
Fibre (%)	3.31	3.39	4.29	3.31	3.39	4.29
Calcium (%)	1.66	1.66	1.66	1.66	1.66	1.66
Phosphorus (%)	0.58	0.59	0.56	0.58	0.59	0.56
Lysine (%)	1.54	1.31	1.28	1.54	1.31	1.28
Methionine (%)	0.61	0.60	0.59	0.61	0.60	0.59

^a^ Starter premix/kg diet: antioxidant 0.125 g; cobalt 0.024 mg; selenium 0.24 mg; iodine 0.014 g; copper 0.006 g; iron 0.024 g; zinc 0.06 g; manganese 0.096 g; chloride 0.05 g; choline chloride 0.05 g; biotin 0.08 mg; cobalamine 0.05 g; niacin 40 mg; pyridoxine 4 mg; riboflavin 6 mg; thiamine 2 mg; Diet 1 (Opt)—optimal energy − protein; Diet 2 (Med)—medium energy − protein; Diet 3 (Low)—low energy–protein; Diet 4 (Opt)—optimal energy − protein + 4 g/kg organic acid; Diet 5 (Med)—medium energy − protein + 6 g/kg organic acids; Diet 6 (Low)—low energy–protein + 8 g/kg organic acids.

**TABLE 2:** Gross composition (%) of experimental diets (finisher 5–8 weeks).

	Diet					
Ingredient	1 Opt	2 Med	3 Low	4 +4 g/kg	5 +6 g/kg	6 +8 g/kg
Maize	56.00	56.00	50.00	56.00	56.00	50.00
Fishmeal	2.00	2.00	2.00	2.00	2.00	2.00
Soya meal	21.50	18.00	14.00	21.50	18.00	14.00
Groundnut cake	8.00	7.00	6.00	8.00	7.00	6.00
Palm oil	2.00			2.00		
Wheat offal	5.00	11.50	22.50	5.00	11.50	22.50
Bonemeal	2.50	2.50	2.50	2.50	2.50	2.50
Oyster shell	2.00	2.00	2.00	2.00	2.00	2.00
Lysine	0.20	0.20	0.20	0.20	0.20	0.20
Methionine	0.30	0.30	0.30	0.30	0.30	0.30
Premix^a^	0.25	0.25	0.25	0.25	0.25	0.25
Salt (NaCL)	0.25	0.25	0.25	0.25	0.25	0.25
Total	100.00	100.00	100.00	100.00	100.00	100.00
Calculated analysis						
M.E (kcal/kg)	3005.80	2871.20	2728.20	3005.80	2871.20	2728.20
CP (%)	21.20	19.22	17.13	21.20	19.22	17.13
Fat (%)	4.54	3.80	3.77	4.54	3.80	3.77
Fibre (%)	3.04	3.30	3.93	3.04	3.30	3.93
Calcium (%)	1.46	1.46	1.46	1.46	1.46	1.46
Phosphorus (%)	0.49	0.48	0.47	0.49	0.48	0.47
Lysine (%)	1.23	1.13	1.01	1.23	1.13	1.01
Methionine (%)	0.62	0.61	0.59	0.62	0.61	0.59

^a^ Finisher premix/kg diet: antioxidant 0.125 g; cobalt 0.25 g; selenium 0.24 g; iodine 0.0014 g; copper 0.006 g; zinc 0.03 g; manganese 0.006 g; choline chloride 0.2 mg; biotin 0.08 mg; folic acid 0.5 mg; pantothenic acid 5.0 mg; cobalamine 0.05 mg; niacin 20 mg; pyridoxine 4.0 mg; riboflavin 3.0 mg; thiamine 2.0 mg; vitamin K 2.5 mg; vitamin E 201.U.; vitamin D3 12; 0001.U.; vitamin A 10; 0001.U. Diet 1 (Opt)—optimal energy − protein; Diet 2 (Med)—medium energy − protein; Diet 3 (Low)—low energy–protein; Diet 4 (Opt)—optimal energy − protein + 4 g/kg organic acid; Diet 5 (Med)—medium energy − protein + 6 g/kg organic acids; Diet 6 (Low)—low energy–protein + 8 g/kg organic acids.

### 2.4 Data Collection

#### 2.4.1 Performance Characteristics

Body weights and feed intake were recorded in each replicate weekly using a digital weighing scale. The weight gain was taken as the difference in the weights of the birds in each successive week. The feed conversion ratio was calculated as the ratio of the feed consumed and weight gain.

#### 2.4.2 Collection of Excreta and Feed Samples for Digestibility Trial

At the starter and finisher phases, two chickens in each replicate with weights close to the average weights in each replicate were chosen for digestibility studies. The chickens had a 2‐day acclimation phase in clean and sterile metabolic cages and underwent a 1‐day fast to empty their gastrointestinal tracts and a 3‐day fresh excreta sample collection. Fresh excreta samples were taken every 24 h repeatedly from pans placed beneath the metabolic cages on days 26–28 and days 54–56. The daily excreta samples were pooled on a replicate‐cage basis, and 100 g samples were obtained and dried in an oven at a temperature of 65°C until constant weights were achieved. The samples were then ground to pass through a 0.5 mm sieve for further analysis of nitrogen‐free extract (NFE), ash, crude fibre, dry matter (DM), CP and ether extract (EE) following the description of AOAC International (2005). The Pauzenga equation (ME (kcal/kg) = 37 CP + 81.1 EE + 35.5 NFE) was used to determine the ME.

NFE was calculated as follows: %NFE = 100 − (%CF + %CP + %EE + %Ash).

#### 2.4.3 Chemical Analyses of Excreta and Feed Samples

The excreta and feed samples were dried at 105°C using a pre‐weighed dried crucible in a convection oven till constant weight was attained to determine the DM (AOAC International 2005, technique no. 930.15). The complete combustion method (AOAC International 2005, method no. 968.06) was used to determine the CP of the excreta and feed samples. The Soxhlet extraction method was used to analyse the EE/fat content of samples (AOAC International 2005, technique no. 99136).

The digestibility of each of the nutrients was calculated as follows:

$$\begin{array}{ccc} & & \mathrm{Nutrient}\;\mathrm{digestibility}\left(\%\right)=\\ & & \frac{\;\mathrm{Nutrient}\;\mathrm{intake}\;\left(\mathrm{gDM}\right)\;-\;\mathrm{Nutrient}\;\mathrm{in}\;\mathrm{excreta}\left(\mathrm{gDM}\right)}{\mathrm{Nutrient}\;\mathrm{intake}\;\left(\mathrm{gDM}\right)\;}\;\times \;100 \end{array}$$

### 2.5 Blood Parameters

Blood samples were collected at 4 and 8 weeks by puncturing the brachial vein from two chickens from each replicate. The first 2.5 mL of the sample was collected into universal anticoagulant‐free bottles for serum analysis, and the remaining 2.5 mL was placed into vials containing ethylenediaminetetraacetate (EDTA) as an anticoagulant for the evaluation of haematological indices. The cyanmethaemoglobin technique was used to calculate the haemoglobin concentration (Hb) (Zijlstra 1997). The Wintrobe haematocrit tube was used to calculate white blood cell, red blood cell counts and packed cell volume using the Schalm et al. (1975) method. On blood smears stained with the May–Grunwald–Giemsa stain, differential leucocyte counts were performed. By centrifuging blood, serum was extracted and stored in the freezer until it was required for biochemical analysis. The amount of albumin and total serum protein was measured using the bromocresol purple technique (Varley et al. 1980). The serum uric acid was calculated using Wootton's (1964) recommended methods. The method outlined by Tietz (1986) was used to measure the serum creatinine level. Alkaline phosphatase, aspartate aminotransferase and alanine aminotransferase were measured using Randox test kits (Randox Laboratory, UK) following normal procedures.

A blood sample (2.5 mL/bird) was collected at 4 and 8 weeks of age by cardiac puncture for lipid profile studies. Blood was collected in clean anticoagulant glass vials. The serum used to determine the total cholesterol (TC), high‐density lipoprotein cholesterol (HDL‐C), total triglycerides (TG) and low‐density lipoprotein cholesterol (LDL‐C) was obtained from blood samples that had been allowed to clot and centrifuged for 20 min. The analysis was carried out using available kits (Randox, UK).

The formula below was used to calculate the values of very low‐density lipoprotein‐cholesterol (VLDL‐C), as described by Lee et al. (2016).

### 2.6 Carcass Characteristics

VLDL−C=TG/5

On Day 56, three chickens from each replicate that represented the average live weights of the replicate group were selected, weighed and sacrificed to determine the following parameters: dressed weight, eviscerated weight, plucked weight, head, shank, thigh, wing, drumstick, back, breast, neck, intestine, gizzard, spleen, abdominal fat and lungs were recorded and calculated as the percentage of live body weights, as described by Oni et al. (2024).

### 2.7 Gut Morphology

Two birds per replicate were chosen for intestinal morphology examinations on Day 56. Meckel's diverticulum was chosen as a reference point, and a 2 cm section of the jejunum was removed, cleaned in sterile water and then fixed in 10% neutral buffered formalin. The tissues were washed under running water to remove excessive fixation before being dehydrated using alcohol in varying concentrations. Pure xylene was used as a clearing agent to remove the dehydrating agent from the tissues, and melted paraffin wax was injected as an embedding agent. The jejunal tissues were then embedded in paraffin blocks, and each sample's paraffin block had 62 mm‐long sections cut out of it using a microtome. Each block's parts were separated by a space of 100 m. After being mounted on glass slides for histological investigation, three of the six 2 m slices were stained with haematoxylin and eosin. Periodic Acid‐Schiff (PAS) stain was used to stain the remaining sections to count the goblet cells per villus. The section was examined using a digital imaging analysis system (Leica DC200, Germany) and a fluorescence microscope (Leica DMLB, Germany). Using an image analysis program (Leica QWin Standard, Version 2.8, Germany), the muscularis layer thickness, crypt depth, villus diameter and villus height were measured as five replications per slice (15 measurements per bird). In the middle 100 m of the PAS‐stained sections, the goblet cell number for each villus was manually recorded (Ahsan et al. 2016).

### 2.8 Gut Microflora Identification and Bacterial Count

The mid portions of the ceca, ileum, jejunum and duodenum were cut into sections that were about 3 cm long (including the digesta) after the gut from the base of the gizzard down to the rectum was dissected. The counting was done using the description of Youssef et al. (2024). Before being freeze‐dried, samples were first snap‐frozen by submersion in liquid nitrogen and held at 20°C. Following each bird's dissection, the dissecting tools were cleaned with 70% ethanol.

Using a modified version of a proprietary extraction technique created by the South Australian Research and Development Institute, total nucleic acid was recovered from chicken intestinal samples. Prior to using the SARDI extraction technique, gut samples were incubated in an adjusted extraction buffer, 65 M phosphate buffer (pH 8.0), 3.4% (wt/vol) *N*‐lauroylsarcosine and 1.7% (wt/vol) polyvinylpyrrolidone at 70°C for an hour.

### 2.9 Statistical Analysis

In a completely randomized design, the data obtained were subjected to a one‐way analysis of variance. When significant, differences among treatment means were separated with the use of Tukey's test as contained in the SAS Institute (2009) package at *p* < 0.05.

## 3 Results

### 3.1 Growth Performance of Broiler Chicks (Starter Phase 0–4 Weeks)

The growth of chicks offered diets of different nutritional planes supplemented with or without organic acids from Day 1 to 28 is shown in Table 3. The feed conversion ratio and feed intake were influenced (*p <* 0.05). Chicks fed a low nutritional plane diet (Treatment 3) without organic acid recorded a higher (*p <* 0.05) total and daily feed intake compared to those fed diets containing an optimal diet with and without organic acids (Treatments 1 and 4). Chicks fed a low nutritional plane diet (Treatment 3) without organic acid recorded a higher (*p <* 0.05) FCR than those fed an optimum diet (Treatment 1 and 4).

**TABLE 3:** Growth performance of chicks (starter phase 0–4 weeks).

Parameter	T1 Opt	T2 Med	T3 Low	T4 Opt 4 g/kg	T5 Med 6 g/kg	T6 Low 8 g/kg	SEM	*p* value
Initial weight (kg)	0.042	0.042	0.042	0.042	0.042	0.042	0.000	
Final weight (kg)	0.575	0.570	0.560	0.580	0.583	0.570	0.004	0.615
Total weight gain (kg)	0.535	0.530	0.520	0.540	0.542	0.530	0.004	0.615
Daily weight (kg)	0.019	0.019	0.019	0.019	0.019	0.019	0.000	0.615
Total feed intake (kg)	1.013^b^	1.068^ab^	1.138^a^	0.983^b^	1.063^ab^	1.092^ab^	0.017	0.016
Daily feed intake (kg)	0.036^b^	0.038^ab^	0.041^a^	0.035^b^	0.038^ab^	0.039^ab^	0.001	0.016
FCR	1.895^b^	2.015^ab^	2.189^a^	1.823^b^	1.963^ab^	2.060^ab^	0.040	0.032
Cost of feed (Naira/kg)	167.76	158.81	152.87	172.23	165.53	161.83	1.311	
Cost of feed intake (Naira/kg)	169.857	169.610	173.890	169.216	175.876	176.361	2.063	0.637
Cost of weight (Naira/kg)	317.960	320.002	334.580	314.061	324.981	333.360	5.112	0.637

*Note*: Means having different superscripts are different (*p <* 0.05) on the same row; T1 (Opt)—optimal energy − protein; T2 (Med)—medium energy − protein; T3 (Low)—low energy– protein; T4 (Opt)—optimal energy − protein + 4 g/kg organic acid; T5 (Med)—medium energy − protein + 6 g/kg organic acids; T6 (Low)—low energy–protein + 8 g/kg organic acids.

Abbreviation: FCR, feed conversion ratio.

### 3.2 Growth Performance at the Finisher Phase (4–8 Weeks)

Birds in Treatment 5 (medium diet with organic acid) had higher (*p <* 0.05) daily weight gain and final weights compared to those on a low diet (Table 4). Broiler chickens in Treatments 1, 4 and 6 (optimal diet with and without organic acid and low diet with organic acid) had higher (*p* *< *0.05) final weights than those in Treatment 3. The birds fed the medium diet without organic acid also had lower weight gains than Treatment 5 chickens. Birds in Treatments 2, 3, 5 and 6 had a higher (*p <* 0.05) total and daily feed intake than those in Treatment 4. Birds in Treatment 3 recorded a higher (*p <* 0.05) FCR than the other groups. Birds in Treatment 2 had a higher (*p <* 0.05) FCR than those in Treatments 4 and 5. Chickens in Treatments 1 and 4 recorded a higher (*p <* 0.05) feed cost per chicken compared to other treatments. Birds in Treatment 3 recorded a significantly higher cost of weight gain compared to those in Treatments 2, 3, 5 and 6.

**TABLE 4:** Growth performance of broiler chickens (5–8 weeks) fed diets of different nutritional planes supplemented with or without organic acids.

Parameters	T1 Opt	T2 Med	T3 Low	T4 Opt 4 g/kg	T5 Med 6 g/kg	T6 Low 8 g/kg	SEM	*p* value
Initial weight (kg)	0.575	0.570	0.560	0.580	0.583	0.570	0.004	0.615
final weight (kg)	2.335^ab^	2.278^b^	2.123^c^	2.373^ab^	2.408^a^	2.335^ab^	0.022	0.001
Total weight gain (kg)	1.760^ab^	1.708^b^	1.563^c^	1.793^ab^	1.825^a^	1.765^ab^	0.022	0.001
Daily weight (kg)	0.063^ab^	0.061^b^	0.056^c^	0.064^ab^	0.065^a^	0.063^ab^	0.001	0.001
Total feed intake (kg)	3.305^bc^	3.433^a^	3.470^a^	3.270^c^	3.378^ab^	3.380^ab^	0.018	0.002
Daily feed intake (kg)	0.118^bc^	0.123^a^	0.124^a^	0.117^c^	0.121^ab^	0.121^ab^	0.001	0.002
FCR	1.881^bc^	2.015^b^	2.223^a^	1.826^c^	1.852^c^	1.917^bc^	0.032	0.001
Cost of feed (Naira/kg)	161.36	146.5	139.56	165.84	153.22	148.52	1.858	
Cost of feed intake (Naira/kg)	533.29^a^	502.86^c^	484.27^d^	542.29^a^	517.50^b^	501.99^c^	4.458	0.001
Feed cost/kg weight (Naira/kg)	303.450^ab^	295.219^ab^	310.180^a^	302.749^ab^	283.822^b^	284.737^b^	3.190	0.069

*Note*: Means on the same row having different superscriptsdiffer (*p <* 0.05); T1 (Opt)—optimal energy − protein; T2 (Med)—medium energy − protein; T3 (Low)—low energy– protein; T4 (Opt)—optimal energy − protein + 4 g/kg organic acid; T5 (Med)—medium energy − protein + 6 g/kg organic acids; T6 (Low)—low energy–protein + 8 g/kg organic acid

Abbreviations: FCR, feed conversion ratio; SEM, standard error of means.

### 3.3 Nutrient Digestibility at the Chick Phase (0–4 Weeks)

Table 5 shows the apparent nutrient digestibility of broilers fed different nutritional planes of diets with or without organic acids. The CP, crude fibre and EE digestibility were significantly (*p <* 0.05) influenced by the dietary treatment. Broiler chickens in Treatment 5 (medium diet supplemented with organic acid, respectively) had a higher (*p <* 0.05) CP digestibility than Treatment 3, while others were statistically similar. Chickens in Treatments 4 and 5 (optimal diet supplemented with organic acid and a medium diet supplemented with organic acid, respectively) had a higher (*p <* 0.05) crude fibre digestibility than the other treatments. Broiler chickens in Treatments 1, 2, 4 and 5 recorded a higher EE digestibility than those in Treatments 3 and 6.

**TABLE 5:** Nutrient digestibility of broiler birds (starter phase 0–4 weeks) fed diets of different nutritional planes supplemented with or without organic acids.

Parameter	T1 Opt	T2 Med	T3 Low	T4 Opt 4 g/kg	T5 Med 6 g/kg	T6 Low 8 g/kg	SEM	*p* value
DM	67.07	68.93	67.06	69.45	70.03	67.52	0.694	0.813
CP	68.83^ab^	68.94^ab^	65.84^b^	69.65^ab^	70.61^a^	66.82^ab^	0.585	0.115
CF	38.31^bc^	38.65^bc^	34.30^c^	39.04^a^	39.49^a^	35.26^bc^	0.672	0.050
EE	78.68^a^	78.73^a^	75.50^b^	79.57^a^	80.67^a^	76.22^b^	0.573	0.004
Ash	59.50	60.02	58.15	61.14	61.40	58.62	0.532	0.477

*Note*: Means on the same row having different superscripts differ (*p <* 0.05); T1 (Opt)—optimal energy − protein; T2 (Med)—medium energy − protein; T3 (Low)—low energy– protein; T4 (Opt)—optimal energy − protein + 4 g/kg organic acid; T5 (Med)—medium energy − protein + 6 g/kg organic acids; T6 (Low)—low energy–protein + 8 g/kg organic acid

Abbreviations: ASH, ash; CF, crude fibre; CP, crude protein; DM, dry matter; EE, ether extract; SEM, standard error of means.

### 3.4 Nutrient Digestibility at the Finisher Phase (4–8 Weeks)

Table 6 presents the apparent nutrient digestibility of chickens at 8 weeks as influenced by diets. There was no treatment effect for nutrient digestibility at the finisher phase.

**TABLE 6:** Nutrient digestibility (finisher 5–8 weeks) fed diets of different nutritional planes supplemented with or without organic acids.

Parameter	T1 Opt	T2 Med	T3 Low	T4 Opt 4 g/kg	T5 Med 6 g/kg	T6 Low 8 g/kg	SEM	*p* value
DM	63.04	69.91	72.20	73.00	81.67	77.87	2.681	0.517
CP	90.01	89.86	91.61	91.48	93.85	93.74	0.789	0.647
CF	66.92	73.28	84.46	76.65	84.26	85.76	2.660	0.228
EE	85.00	91.53	91.49	90.31	93.63	92.05	1.082	0.288
Ash	71.03	79.63	77.29	78.84	85.62	83.59	2.046	0.454

*Note*: T1 (Opt)—optimal energy − protein; T2 (Med)—medium energy − protein; T3 (Low)—low energy– protein; T4 (Opt)—optimal energy − protein + 4 g/kg organic acid; T5 (Med)—medium energy − protein + 6 g/kg organic acids; T6 (Low)—low energy–protein + 8 g/kg organic acid

Abbreviations: CF, crude fibre; CP, crude protein; DM, dried matter; EE, ether extract.

### 3.5 Haematological Characteristics at the Starter Phase (0–4 Weeks)

The haematological characteristics of broiler chicks fed diets of different nutritional planes supplemented with or without organic acids from the 28th day are presented in Table 7. Most of the parameters were not affected (*p *> 0.05) by the treatment except PCV, Hb, WBC and MCHC. Birds in Treatments 1 and 5 (optimal diet without organic acids and medium diet with organic acids, respectively) recorded a higher (*p <* 0.05) PCV than those in Treatment 3 (low diet without organic acids). Birds in Treatments 2, 4 and 5 (medium diet without organic acids, optimal diet and medium diet with organic acids, respectively) recorded a higher (*p <* 0.05) Hb compared to those in Treatment 3 (low diet without organic acids). Birds in Treatments 4 and 5 (optimal diet and medium diet with organic acids, respectively) recorded a similar WBC with T2 but higher (*p <* 0.05) than those in T3 and T6 treatments. Birds in Treatments 4 and 6 (optimal diet and low diet with organic acids, respectively) recorded a higher (*p <* 0.05) MCHC than those in Treatment 3 (low diet without organic acids).

**TABLE 7:** Haematology characteristics of broiler chickens (starter 0–4 weeks) fed diets of different nutritional planes supplemented with or without organic acids.

Parameter	T1 Opt	T2 Med	T3 Low	T4 Opt 4 g/kg	T5 Med 6 g/kg	T6 Low 8 g/kg	SEM	*p* value
Packed cell volume (%)	33.00^a^	32.33^ab^	27.67^c^	29.33^bc^	33.00^a^	28.67^bc^	0.681	0.032
Haemoglobin (g/100 mL)	9.20^ab^	9.97^a^	8.57^b^	10.10^a^	10.00^a^	8.93^ab^	0.194	0.061
Red blood cells (×10^6^/mm^3^)	2.70	2.73	2.60	2.83	2.76	2.66	0.065	0.761
White blood cells (×10^3^/mm^3^)	10.87^bc^	12.00^ab^	10.27^c^	12.33^a^	12.23^a^	10.63^bc^	0.251	0.029
Heterophil (%)	27.00	27.67	26.33	32.00	28.33	29.00	0.886	0.568
Lymphocyte (%)	69.67	69.67	64.00	71.00	68.67	68.67	0.984	0.454
Eosinophil (%)	1.00	1.00	0.67	1.33	1.67	1.00	0.196	0.822
Basophil (%)	1.00	1.00	0.67	1.33	0.67	0.67	0.159	0.840
Monocyte (%)	1.00	0.33	1.33	1.33	1.33	0.67	0.140	0.164
MCV (mg/100 mL)	116.80	118.92	119.90	110.95	109.70	113.37	1.606	0.348
MCH (%)	35.75	36.80	36.23	34.83	33.93	35.32	0.488	0.663
MCHC (%)	30.61^ab^	30.93^ab^	30.22^b^	31.37^a^	30.95^ab^	31.17^a^	0.129	0.113

*Note*: Means on the same row having different superscripts differs (*p <* 0.05); T1 (Opt)—optimal diet; T2 (Med)—medium diet; T3 (Low)—low diet; T4 (Opt)—optimal diet with 4 g/kg organic acid; T5 (Med)—medium diet with 6 g/kg organic acids; T6 (Low)—low diet with 8 g/kg organic acids.

### 3.6 Haematological Characteristics at the Finisher Phase (4–8 Weeks)

The haematological characteristics of broiler chicks fed diets of different nutritional planes supplemented with or without organic acids on the 56th day are presented in Table 8. The PCV of the birds in Treatment 3 (low diet without organic acids) was significantly lower (*p <* 0.05) than those of Treatments 1, 4 and 5 (optimal diet without organic acids, optimal diet and medium diet with organic acids). Birds in Treatment 4 (optimal diet with organic acids) recorded a significantly higher (*p <* 0.05) haemoglobin compared to those in Treatment 3 (low diet without organic acids), while the haemoglobin of birds on other treatments did not differ significantly. Birds in Treatment 4 (optimal diet with organic acids) recorded a significantly higher WBC (*p <* 0.05) compared to those in Treatment 3 (low diet without organic acids). The white blood cells of birds on other treatments did not differ significantly. Birds in Treatments 1, 4 and 5 (optimal diet without organic acids, optimal diet and medium diet with organic acids) recorded a significantly higher (*p <* 0.05) LYM % compared to those in Treatment 3 (low diet without organic acids), while the LYM % of birds on other treatments did not differ significantly. Birds in Treatment 4 (optimal diet with organic acids) recorded a significantly higher (*p <* 0.05) MCH compared to those in Treatment 3 (low diet without organic acids). The MCHC of the birds in T3 was lower than those in T5 but comparable to the other treatment groups.

**TABLE 8:** Haematology characteristics of broiler birds (finisher 4–8 weeks) fed diets of different nutritional planes supplemented with or without organic acids.

Parameter	T1 Opt	T2 Med	T3 Low	T4 Opt 4 g/kg	T5 Med 6 g/kg	T6 Low 8 g/kg	SEM	*p* value
Packed cell volume (%)	36.00^a^	34.67^ab^	30.33^b^	38.00^a^	36.33^a^	33.67^ab^	0.817	0.083
Haemoglobin (g/100 mL)	12.22^ab^	11.17^ab^	10.67^b^	12.63^a^	11.97^ab^	33.67^ab^	0.249	0.206
Red blood cells (×10^6^/mm^3^)	3.00	3.10	2.63	3.07	3.27	2.90	0.084	0.401
White blood cells (×10^3^/mm^3^)	15.93^ab^	15.77^ab^	14.03^b^	17.27^a^	15.73^ab^	15.13^ab^	0.368	0.226
Heterophil (%)	30.67	32.62	30.67	31.00	32.00	29.67	1.339	0.110
Lymphocyte%	67.67^a^	65.00^ab^	57.33^b^	66.67^a^	69.00^a^	62.67^ab^	1.323	0.097
Eosinophil (%)	0.67	0.67	0.33	1.33	0.33	0.33	0.164	0.506
Basophil (%)	0.33	0.33	1.00	0.33	0.33	0.33	0.145	0.771
Monocyte (%)	1.00	0.67	1.00	1.00	0.67	1.33	0.221	0.972
MCV (mg/100 mL)	120.10	112.60	116.30	124.93	111.77	115.40	1.947	0.405
MCH (%)	40.87^ab^	39.43^ab^	36.20^b^	41.53^a^	40.70^ab^	36.77^ab^	0.732	0.138
MCHC (%)	33.97^ab^	33.93^ab^	32.20^b^	33.23^ab^	35.20^a^	32.93^ab^	0.334	0.131

*Note*: Means on the same row having different superscripts are significantly different (*p <* 0.05); T1 (Opt)—optimal diet; T2 (Med)—medium diet; T3 (Low)—low diet; T4 (Opt)—optimal diet with 4 g/kg organic acid; T5 (Med)—medium diet with 6 g/kg organic acids; T6 (Low)—low diet with 8 g/kg organic acids.

Abbreviations: MCH, mean corpuscular haemoglobin; MCHC, mean corpuscular haemoglobin concentration; MCV, mean corpuscular volume.

### 3.7 Serum Biochemical Indices and Blood Lipid Profile at the Starter Phase (0–4 Weeks)

The serum biochemistry of broiler chicks fed diets of different nutritional planes supplemented with or without organic acids at 4 weeks of the experiment is presented in Table 9. Most of the parameters were not affected by the treatment. However, birds in Treatment 5 (medium diet with organic acids) recorded a higher [*p <* 0.05] AST compared to those in Treatment 3 [low diet without organic acids], while the AST of birds on other treatments did not differ significantly.

**TABLE 9:** Serum Biochemical indices and lipid profiles of broiler birds (starter 0–4 weeks) fed diets of different nutritional planes supplemented with or without organic acids.

Parameter	T1 Opt	T2 Med	T3 Low	T4 Opt 4 g/kg	T5 Med 6 g/kg	T6 Low 8 g/kg	SEM	*p* value
Total protein, g/dL	3.00	2.97	2.67	3.20	3.20	3.27	0.121	0.698
Albumin (g/dL)	1.80	1.77	1.43	1.96	1.80	1.67	0.082	0.621
Globulin (g/dL)	1.20	1.50	1.23	1.20	1.53	1.30	0.070	0.625
AST (U/L)	35.23^ab^	33.57^ab^	21.93^b^	28.23^ab^	45.10^a^	38.77^ab^	2.844	0.242
ALT (U/L)	9.90	6.33	8.97	9.30	6.33	8.73	0.832	0.779
Uric acid (mg/dL)	12.10	5.93	9.00	7.90	4.30	5.40	1.087	0.358
Triglyceride (mg/dL)	93.97	98.10	120.57	101.87	79.10	127.10	8.202	0.627
Cholesterol (mg/dL)	117.03	113.60	121.77	126.07	119.07	118.80	7.349	0.999
Creatinine (mg/dL)	0.77	0.60	0.63	0.50	0.57	0.70	0.044	0.627
VLDL (mg/dL)	18.80	19.60	25.40	20.40	15.83	24.13	1.640	0.629
HDL (mg/dL)	59.03	54.97	60.03	66.00	60.07	61.80	3.518	0.982
LDL (mg/dL)	31.60	43.17	39.67	37.60	39.03	39.20	3.477	0.976

*Note*: Means on the same row having different superscripts are significantly different (*p <* 0.05); T1 (Opt)—optimal diet; T2 (Med)—medium diet; T3 (Low)—low diet; T4 (Opt)—optimal diet with 4 g/kg organic acid; T5 (Med)—medium diet with 6 g/kg organic acids; T6 (Low)—low diet with 8 g/kg organic acids.

Abbreviations: ALT, alanine aminotransferase; AST, aspartate aminotransferase; HDL, high‐density lipoprotein; LDL, low‐density lipoprotein; VLDL, very low‐density lipoprotein.

### 3.8 Serum Biochemical Indices and Blood Lipid Profile (Finisher 4–8 Weeks)

The serum biochemical parameters and lipid profile of chickens fed diets of different nutritional planes supplemented with or without organic acids at 8 weeks of the experiment are presented in Table 10. The total protein ranged from 4.33 to 5.63, albumin from 2.50 to 3.27, globulin from 1.83 to 2.37, AST from 85.23 to 108.80, ALT from 4.53 to 9.37, uric acid from 9.33 to 12.60, triglyceride from 111.97 to 149.30, cholesterol from 68.23 to 124.40 and creatinine from 0.90 to 1.83. The blood lipid profile of broiler chicks fed diets of different nutritional planes supplemented with or without organic acids at 8 weeks was not influenced by the treatment.

**TABLE 10:** Serum biochemical indices and lipid profiles of broiler birds (finisher 5–8 weeks) fed diets of different nutritional planes supplemented with or without organic acids.

Parameter	T1 Opt	T2 Med	T3 Low	T4 Opt 4 g/kg	T5 Med 6 g/kg	T6 Low 8 g/kg	SEM	*p* value
Total protein (g/dL)	5.16	4.83	4.33	5.63	5.30	4.97	0.269	0.859
Albumin (g/dL)	2.97	2.77	2.50	3.27	3.03	2.90	0.156	0.856
Globulin (g/dL)	2.20	2.07	1.83	2.37	2.27	2.10	0.115	0.872
AST (U/L)	85.23	99.33	86.00	94.27	108.80	105.80	3.770	0.380
ALT (U/L)	4.53	6.27	9.37	5.20	6.90	8.90	0.743	0.359
Uric acid (mg/dL)	12.60	11.60	9.93	9.57	12.30	9.33	0.527	0.407
Triglyceride (mg/dL)	146.50	133.63	140.87	111.97	149.30	145.43	6.520	0.645
Cholesterol (mg/dL)	79.03	100.73	68.23	99.10	124.40	94.33	9.123	0.644
Creatinine (mg/dL)	1.83	1.73	1.10	1.67	1.40	0.90	0.177	0.649
VLDL (mg/dL)	26.73	29.87	28.17	22.40	29.30	29.10	1.302	0.644
HDL (mg/dL)	44.60	32.17	22.93	48.87	57.57	42.60	5.677	0.624
LDL (mg/dL)	27.83	36.97	16.20	20.13	26.83	23.57	3.353	0.637

*Note*: Means on the same row, having different superscripts are significantly different (*p <* 0.05); T1 (Opt)—optimal diet; T2 (Med)—medium diet; T3 (Low)—low diet; T4 (Opt)—optimal diet with 4 g/kg organic acid; T5 (Med)—medium diet with 6 g/kg organic acids; T6 (Low)—low diet with 8 g/kg organic acids.

Abbreviations: ALT, alanine aminotransferase; AST, aspartate aminotransferase; HDL, high‐density lipoprotein; LDL, low‐density lipoprotein; VLDL, very low‐density lipoprotein.

### 3.9 Gut Morphometry

The gut (jejunum) morphometry of broiler birds fed diets of different nutritional planes supplemented with or without organic acids is presented in Table 11. The birds in Treatments 1, 2, 4 and 5 (optimal diet without organic acids, medium diet without organic acids, optimal diet and medium diet with organic acids) recorded a significantly higher (*p <* 0.05) basal width compared to those in Treatment 3 (low diet without organic acids). The treatment did not have an impact on the other parameters measured.

**TABLE 11:** Gut (jejunum) morphometry of broiler birds fed diets of different nutritional planes supplemented with or without organic acids.

Parameter	T1 Opt	T2 Med	T3 Low	T4 Opt 4 g/kg	T5 Med 6 g/kg	T6 Low 8 g/kg	SEM	*p* value
Villus height (mm)	529.00	475.00	469.00	660.00	745.00	582.00	49.111	0.634
Apical width (mm)	40.00	35.00	35.00	35.00	40.00	35.00	1.930	0.777
Basal width (mm)	95.00^a^	90.00^a^	70.00^b^	90.00^a^	95.00^a^	76.00^ab^	3.516	0.150
Laminal proprial depth (mm)	157.50	124.00	166.00	173.00	163.00	247.50	17.284	0.531

*Note*: Means having different superscripts differ (*p <* 0.05) on the same row; T1 (Opt)—optimal diet; T2 (Med)—medium diet; T3 (Low)—low diet; T4 (Opt)—optimal diet with 4 g/kg organic acid; T5 (Med)—medium diet with 6 g/kg organic acids; T6 (Low)—low diet with 8 g/kg organic acids.

### 3.10 Microbial Counts

In comparison to the birds in Treatment 3 (low diet), total bacteria count was significantly (*p <* 0.05) reduced in Treatment 5 (medium diet), while bacterial count in birds of other treatments was not significantly different (Table 12).

**TABLE 12:** Microbial count of broiler chickens fed diets of different nutritional planes supplemented with or without organic acids.

Parameter	T1 Opt	T2 Med	T3 Low	T4 Opt 4 g/kg	T5 Med 6 g/kg	T6 Low 8 g/kg	SEM	*p* value
TBC	1.65^ab^	1.25^ab^	2.45^a^	1.55^ab^	1.20^b^	1.75^ab^	0.159	0.232
*Escherichia coli*	+++	+−+	+++	++−	+−	++−		
*Enterobacter* spp.	++−	−+−	+++	−+	−−	+−+		
*Streptococcus* spp.	+++	+++	+−	+++	+++	+−+		
*Staphylococcus* spp.	+++	+++	+++	+−+	+−+	++−		
*Pseudomonas* spp.	+−	−−	++−	+−	−−	+−		

*Note*: Means having different superscripts differ (*p <* 0.05) on the same row; TBC—total bacteria count × 10^6 ^Cfu/g; (−)Absence of bacteria; (+)Presence of bacteria; T1 (Opt)—optimal diet; T2 (Med)—medium diet; T3 (Low)—low diet; T4 (Opt)—optimal diet with 4 g/kg organic acid; T5 (Med)—medium diet with 6 g/kg organic acids; T6 (Low)—low diet with 8 g/kg organic acids.

### 3.11 Carcass Characteristics

The carcass characteristics of broiler chickens fed diets of different nutritional planes supplemented with or without organic acids at 8 weeks are shown in Table 13. Most of the parameters were not significantly (*p <* 0.05) influenced by the treatment. Birds in Treatment 5 (medium diet with organic acids) recorded a higher (*p <* 0.05) eviscerated and dressed percentage compared to those in Treatments 2 and 3 (medium diet and low diet without organic acids). The eviscerated and dressed percentages of birds in the other treatments did not differ significantly. Broiler chickens in Treatments 5 and 6 (medium diet and low diet with organic acids) recorded a higher (*p <* 0.05) relative wings’ weight compared to those in Treatment 3 (low energy—low protein without organic acids). The relative wings’ weights of birds on other treatments were similar. Broiler chickens in Treatment 4 (optimal diet with organic acids) recorded a higher (*p <* 0.05) relative lung weight compared to those in Treatment 3 (low diet without organic acids). However, there was no difference in the relative lung weights of the chickens of other treatment groups.

**TABLE 13:** Carcass traits of broilers fed diets of suboptimal nutritional planes supplemented with or without organic acids.

Parameter	T1 Opt	T2 Med	T3 Low	T4 Opt 4 g/kg	T5 Med 6 g/kg	T6 Low 8 g/kg	SEM	*p* value
Body weight (kg)	2.133	2.133	2.100	2.143	2.150	2.125	0.24	0.949
Plucked (%)	93.80	91.47	92.06	92.82	93.687	92.60	0.70	0.949
Eviscerated (%)	80.45^ab^	79.99^b^	79.36^b^	83.48^ab^	86.60^a^	83.60^ab^	0.91	0.140
Dressed (%)	75.76^ab^	72.92^b^	73.78^b^	78.70^ab^	81.03^a^	78.68^ab^	0.93	0.049
Head (%)	2.83	2.85	2.84	2.82	3.07	2.67	0.06	0.688
Shank (%)	3.91	3.80	3.54	3.54	4.07	3.89	0.15	0.913
Thigh (%)	9.66	11.18	9.93	10.70	11.40	11.06	0.28	0.423
Wing (%)	9.52^ab^	9.20^ab^	8.84^b^	9.86^ab^	10.64^a^	10.87^a^	0.25	0.080
Drumstick (%)	10.09	10.69	10.56	10.25	10.78	11.06	0.16	0.625
Back (%)	12.75	12.48	11.81	12.82	12.71	12.84	0.27	0.919
Breast (%)	20.10	22.70	21.33	22.19	23.09	21.41	0.63	0.836
Neck (%)	5.02	4.46	4.92	4.93	4.37	4.76	0.13	0.675
Intestine (%)	5.28	5.09	5.16	4.41	4.90	4.27	0.24	0.826
Gizzard (%)	2.08	1.99	1.96	1.94	2.17	2.04	0.08	0.976
Abdominal fat (%)	0.78	0.96	0.78	1.08	0.97	1.25	0.10	0.813
Lungs (%)	0.57^ab^	0.54^ab^	0.44^b^	0.62^a^	0.54^ab^	0.53^ab^	0.02	0.320
Spleen (%)	0.10	0.09	0.42	0.08	0.09	0.06	0.06	0.463

*Note*: Means on the same row having different superscripts are significantly different (*p <* 0.05); T1 (Opt)—optimal diet; T2 (Med)—medium diet; T3 (Low)—low diet; T4 (Opt)—optimal diet with 4 g/kg organic acid; T5 (Med)—medium diet with 6 g/kg organic acids; T6 (Low)—low diet with 8 g/kg organic acids.

Abbreviation: SEM, standard error of means.

## 4 Discussion

The study evaluated the responses of broilers to diets of suboptimal nutritional plane supplemented with or without organic acid. The enhanced FCR in the chickens fed diets supplemented with organic acids from 4 to 8 weeks compared to those fed diets without organic acids could be attributed to improved nutrient utilization, culminating in higher body weights. This aligns with the report of Azza and Ragaa (2014). Parks et al. (2001) indicated that organic acid supplementation enhanced FCR owing to reduced feed consumption and higher weight gain, in addition to improved gut health, leading to improved nutrient utilization, absorption and digestion. Our observation is in agreement with the reports of Ghazalah et al. (2011).

The higher weight gain and feed efficiency of the birds fed diets with organic acids in feeds in this study align with the report of Owens et al. (2008), Hudha et al. (2010) and Sheikh et al. (2011), which indicated that the chickens’ body weights were positively affected by the dietary organic acid supplementation. Zulfqarul et al. (2017) observed that there is a multiplication of pathogenic bacteria in the gut, causing cell proliferation and thickening of the intestinal membrane and also damaging the villus, thereby making the absorption of nutrients difficult, leading to a decreased growth rate. Supplementation of organic acids could improve the chickens’ performance by decreasing the pathogenic bacteria population (Khan and Iqbal 2016). The antimicrobial actions (Paul et al. 2007), gastrointestinal tract pH modulation (Dibner 2004) and stimulation of endogenous secretions like bile (Thaela 1998) of organic acids have been documented. Several studies indicate that citric acid inclusion in the diets of broilers enhanced growth performance parameters (Afsharmanesh and Pourreza 2005; Abdel‐Fattah et al. 2008). Lima et al. (2008) also reported that carcass yield could be improved with an increase in dietary CP, followed by dietary energy, respectively. The carcass yield was observed to be significantly lower in Treatment 3 (low diet without organic acid). They indicate that the nutrient content was inadequate for the chickens.

The cost of production was lowest in Treatments 6 and 5 (low diet and medium diet with organic acids) compared with those without organic acids during the finisher phase in the present trial. Similar findings had earlier been documented by Venkatasubramani et al. (2014) and Khaidem et al. (2019), who reported a higher profit margin due to the supplementation of organic acid. The observed low cost of production per kilogram in Treatments 5 and 6 in this study is an indication that the inclusion of dietary organic acid increased nutrient digestibility and utilization.

A better digestibility of protein was observed in Treatments 4 and 5 (optimal diet and medium diet with organic acids) compared with those without organic acids during the starter phase. Organic acids possess the capacity to reduce the bacteria population in the gut, resulting in better nutrient digestibility (Canibe et al. 2008). This corresponds to the improved performance in body weights recorded in the chickens offered dietary organic acids. This observation agrees with the findings of Ndelekwute et al. (2015), who indicated that the inclusion of organic acid enhanced the crude fibre, CP and EE digestibility. Kemme (1998) also reported an improvement in crude fibre, EE and CP as well as an improvement in DM digestibility, which is in contrast to the results in this study. The similarity in the nutrient digestibility parameters at the finisher phase is in contrast to the observation of Hernandez, Madrid, et al. (2006).

The higher dressed weights and eviscerated weights in birds offered dietary organic acids (T5) than those without organic acids in the present study is similar to the findings of Aksu et al. (2007), Hudha et al. (2010), Fascina et al. (2012) and Khaidem et al. (2019), who recorded increased carcass yields and dressing percentages due to dietary organic acid supplementation in broilers. The wing parameters were low in Treatment 3 (low diets without organic acid) compared to Treatments 5 and 6 (medium and low diets supplemented with organic acids). Similar to our findings, Mendes et al. (2004) observed that nutritional increase (ME and lysine) improved the wing yield, which is in line with what was observed in treatments supplemented with organic acids (4, 5 and 6). The increase in wing yield as nutritional level increases observed in treatments without organic acids conforms with the observation of Muñoz et al. (2018), who also observed an increase in wing yield as nutritional level increases. The lack of statistical difference in the other carcass parameters across treatment groups in this study aligns with the earlier results of Thirumeignanam et al. (2006).

The increase in white blood cells in chickens treated with organic acids supplementation in this trial agrees with the observation of Wang et al. (2010), who recorded an increase in WBC in layers fed organic acids compared to those without organic acids. Cetin et al. (2006) also indicated a higher level of Hb in layers supplemented with humic acid. Banaszkiewick and Drobnik (1994) revealed that haemoglobin and PCV increased in rats treated with humic acid. A high PCV above the normal range is an indicator of toxic factors which could have detrimental effects on blood formation in birds. The level of AST was higher in organic acid groups than those without organic acids at the starter phase. The results align with the report by Ndelekwute et al. (2018) that AST levels increased in the birds fed butyric and acetic acid.

The similarity of the serum biochemical parameters of the birds obtained during the finisher phase agrees with the findings of Hernandez, Garcia, et al. (2006) that organic acid supplementation had no effect on the blood metabolites in broilers. The lack of difference in the blood lipid profile obtained during the starter and finisher phases in the present trial aligns with the findings of Yesilbag and Colpan (2006), which indicate that supplementation of organic acids did not influence the blood lipid profile of hens.

The higher basal width in broilers fed diets of different nutritional planes supplemented with organic acid agrees with the report of Leeson et al. (2005) and Panda et al. (2009), who observed that different dietary butyric acid inclusion levels enhanced the basal width. The improvement in basal width in broiler chickens may be explained by the fact that organic acids inhibit the development of a variety of pathogens in the intestine. This inhibits the process of infection, which in turn inhibits inflammatory responses in the intestinal mucosa (Loddi et al. 2004; Pelicano et al. 2005).

The lower total bacteria count recorded in broiler chickens offered a diet of different nutritional planes supplemented with organic acids indicates that organic acid reduced the gastrointestinal microbial population. This conforms to the findings of Ndelekwute et al. (2018) that organic acids have the ability to reduce the total bacteria count. The reduction of gram‐negative bacteria like *Pseudomonas* spp.*, Enterobacter* spp. and *Escherichia coli* in the treatments with organic acids when compared with those without organic acids has also been documented (Ndelekwute et al. 2018; Russell and Diez‐Gonzalez 1998). Sun (2004) reported that organic acids are an excellent antibacterial agent in the gut. *Salmonella enteritidis* has been shown to exhibit less virulence and invasiveness when treated with butyric acid, which results in less colonization of the caeca of broiler chickens (Van Immerseel et al. 2006).

To conclude, in the starter phase, the supplementation of organic acid in an 8 g/kg diet was beneficial to the feed conversion ratio of the birds offered a low‐energy and low‐protein diet. Moreover, the addition of organic acid in a 6 g/kg diet to the medium‐energy and medium‐protein diet and also an 8 g/kg diet to a low‐energy and low‐protein diet enhanced the feed conversion ratio, final body weights and cost of production during the finisher phase. The inclusion of organic acid in the suboptimal nutritional plane diets did not elicit a consistent response in the total bacteria count and the bacteria load of broiler chickens. Supplementation of medium energy—medium protein with 6 g/kg of organic acids is recommended for improved performance, carcass characteristics, blood parameters and microbial count of broiler chickens. Supplementation of a low‐energy and low‐protein diet with 8 g/kg of organic acids also enhanced the performance of the broilers while reducing the cost of production per weight gain.

## Author Contributions

**Kolade Ogunola**: data curation, formal analysis, funding acquisition, investigation, methodology, project administration, resources, software, validation, visualization, writing – original draft, writing – review and editing. **A. V. Jegede**: conceptualization, data curation, formal analysis, funding acquisition, investigation, methodology, project administration, resources, software, supervision, validation, visualization, writing – original draft, writing – review and editing. **Adeboye Fafiolu**: conceptualization, data curation, formal analysis, funding acquisition, investigation, methodology, project administration, resources, software, supervision, validation, writing – original draft, writing – review and editing. **Oyegunle Emmanuel Oke**: conceptualization, data curation, formal analysis, funding acquisition, investigation, methodology, project administration, supervision, validation, visualization, writing – original draft, writing – review and editing

## Conflicts of Interest

The authors declare no conflicts of interest.

## Ethics Statement

The trial was carried out in line with the Institutional Animal Ethics Committee guidelines of the Federal University of Agriculture, Abeokuta, Nigeria. The chickens were given adequate management and care without unnecessary discomfort during the study.

### Peer Review

The peer review history for this article is available at https://www.webofscience.com/api/gateway/wos/peer‐review/10.1002/vms3.70332

## Data Availability

The datasets generated during and/or analysed during the current study are not publicly available because they are part of ongoing studies but are available from the corresponding author on reasonable request.
